# Supplementary material for: Open-channel block of human TRPV6 by polyamine spermine
Source: Nat Commun. 2026 May 27;17:4720. doi: 10.1038/s41467-026-73653-5 (PMC13216613; doi:10.1038/s41467-026-73653-5)
Supplement: Supplementary file 1 — Supplementary Information [file 41467_2026_73653_MOESM1_ESM.pdf]

Supplementary Information

Open-channel block of human TRPV6 by polyamine spermine

Arthur Neuberger[1,7], Irina I. Veretenenko[2,4,7], Alexey Shalygin[3,7], Yury A. Trofimov[2,4], Thomas Gudermann[3,5], Vladimir Chubanov[3,#], Roman G. Efremov[2,4,6,#], Alexander I. Sobolevsky[1,#]

[1] Department of Biochemistry and Molecular Biophysics, Columbia University, New York 10032, NY, USA

[2] Shemyakin-Ovchinnikov Institute of Bioorganic Chemistry, Russian Academy of Sciences, 117997 Moscow, Russia.

[3] Walther-Straub Institute of Pharmacology and Toxicology, LMU Munich, 80336 Munich, Germany.

[4] Research Institute for Systems Biology and Medicine, Moscow, 117246 Moscow, Russia.

[5] Comprehensive Pneumology Center, German Center for Lung Research, 81377 Munich, Germany.

[6] National Research University Higher School of Economics, 101000 Moscow, Russia.

[7] These authors contributed equally: Arthur Neuberger, Irina I. Veretenenko, Alexey Shalygin.

[#] Correspondence: vladimir.chubanov@lrz.uni-muenchen.de; efremov@nmr.ru; as4005@cumc.columbia.edu

This PDF file includes:

Supplementary Figures 1-14

Supplementary Tables 1-2

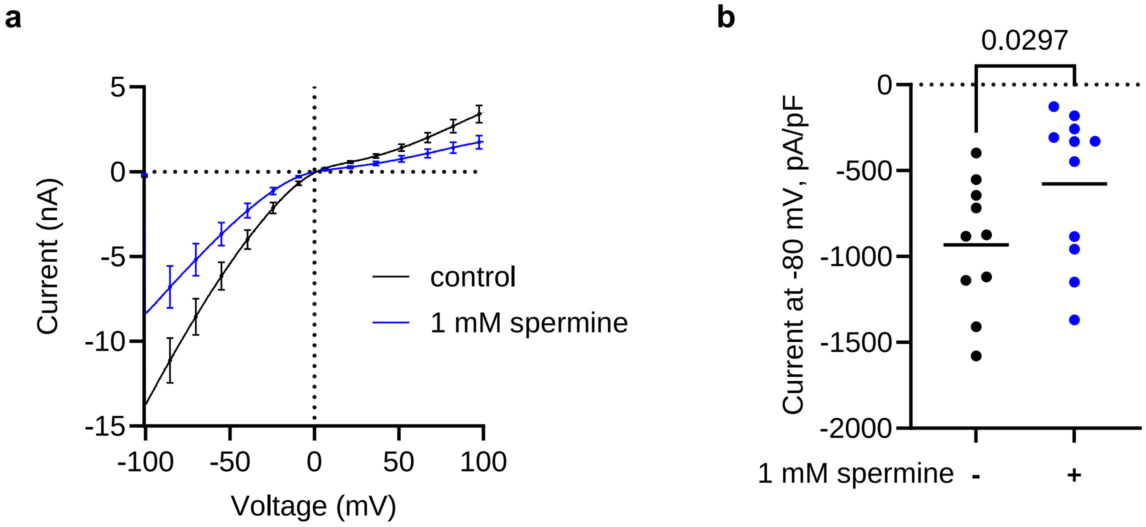

Supplementary Figure 1. Effect of extracellular spermine on the TRPV6 Na^{+} currents. **a**, Voltage dependence of whole-cell sodium currents (mean ± SEM) recorded from TRPV6-expressing HEK 293 cells in the absence (black, n = 10) or presence (blue, n = 11) of 1 mM extracellular spermine using the ramp protocol shown in Fig. 1d. **b,** Current amplitudes measured at −80 mV in the experiment illustrated in (**a**). Data are normalized to the cell size as pA/pF and shown as mean; *p* values are shown for the unpaired one-tailed *t*-test. Source data are provided.

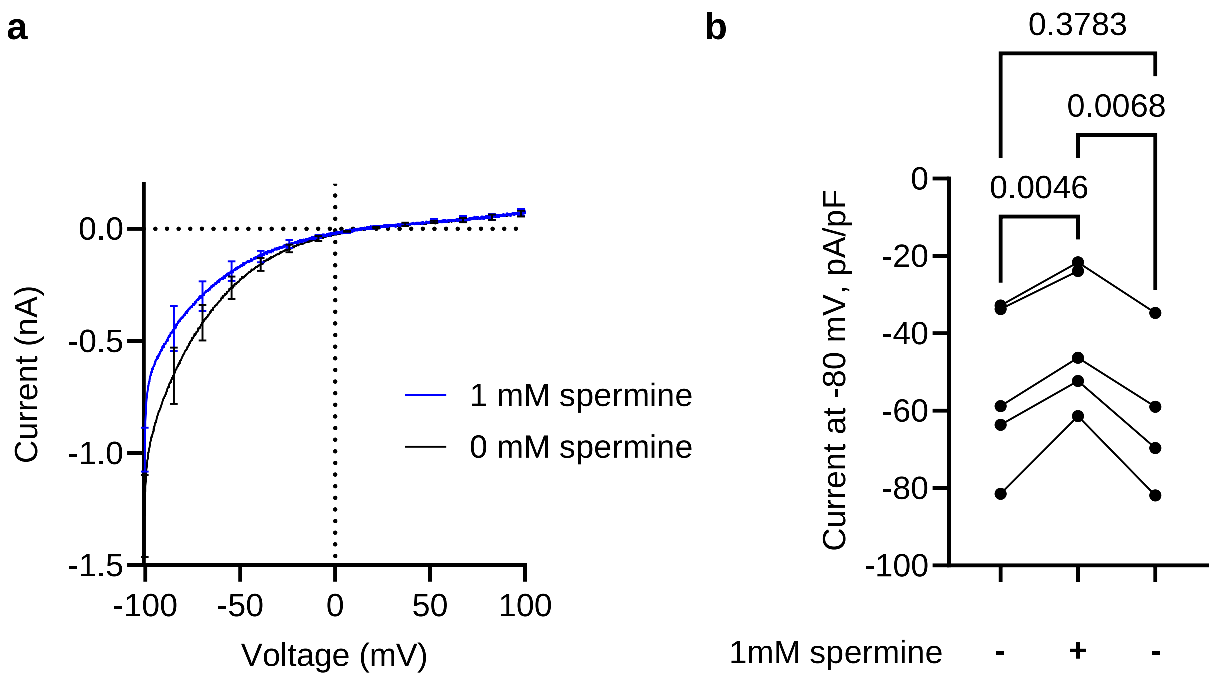

Supplementary Figure 2. Effect of extracellular spermine on the TRPV6 Ca^{2+} currents. a, Voltage dependence of whole-cell Ca^{2+} currents (mean ± SEM) recorded from TRPV6-expressing HEK 293 cells in the absence (black) or presence (blue) of 1 mM extracellular spermine using the ramp protocol shown in Fig. 1d. **b,** Current amplitudes measured at −80 mV in the experiment illustrated in (**a**). Data are normalized to the cell size as pA/pF. Lines connect the data points from individual experiments with spermine application (n = 5) and washout (where applicable, n = 4); p-values are shown for Tukey's multiple comparisons test. Source data are provided.

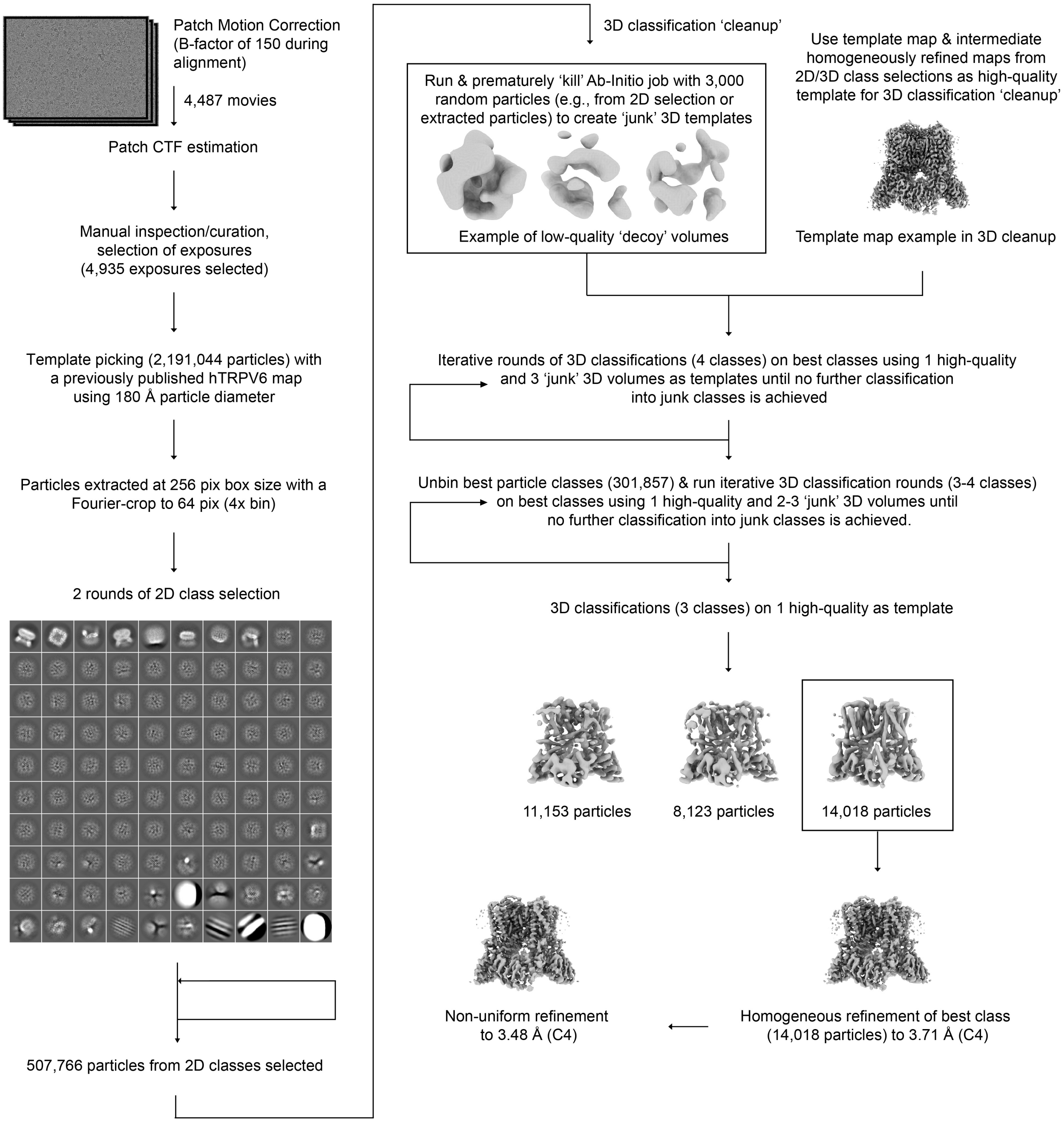

Supplementary Figure 3. 3D reconstruction workflow for hTRPV6$_{SPM}$. Examples of micrographs, 2D class averages and 3D volumes are shown at different stages of cryo-EM data processing workflow.

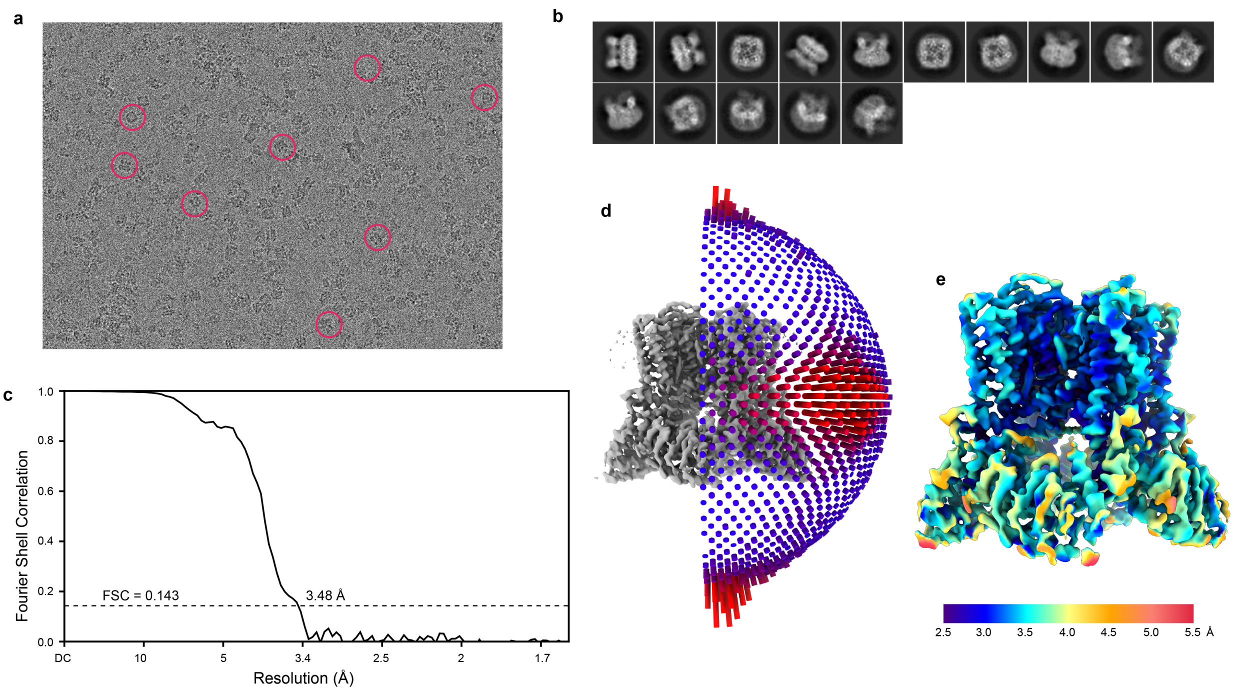

Supplementary Figure 4. Overview of cryo-EM for hTRPV6$_{SPM}$. a, Representative micrograph with example particles circled in red. **b**, 2D class averages. **c**, FSC curve. **d**, Euler angle distribution of particles contributing to final reconstructions of hTRPV6$_{SPM}$, with larger red cylinders representing orientations comprising more particles. **e**, Local resolution presented as coloring of the hTRPV6$_{SPM}$ cryo-EM map.

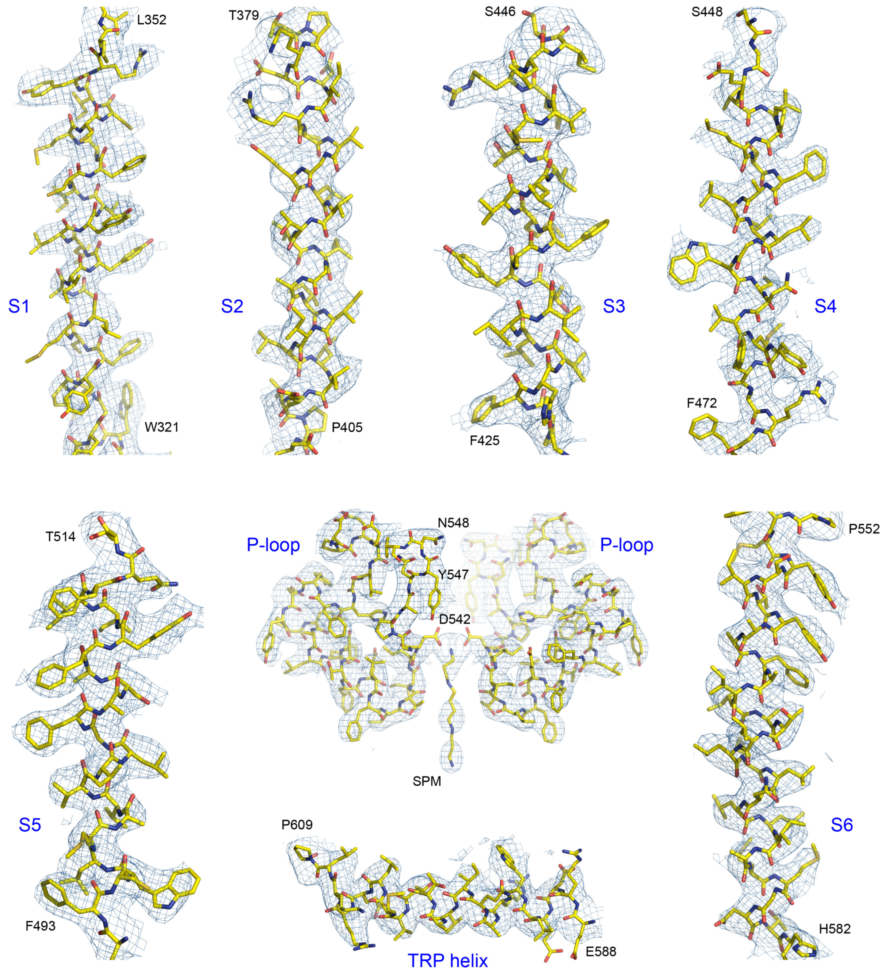

Supplementary Figure 5. Cryo-EM density for hTRPV6$_{SPM}$. Fragments of the TMD for hTRPV6$_{SPM}$, with the structural model shown as yellow sticks and the corresponding cryo-EM density as a blue mesh.

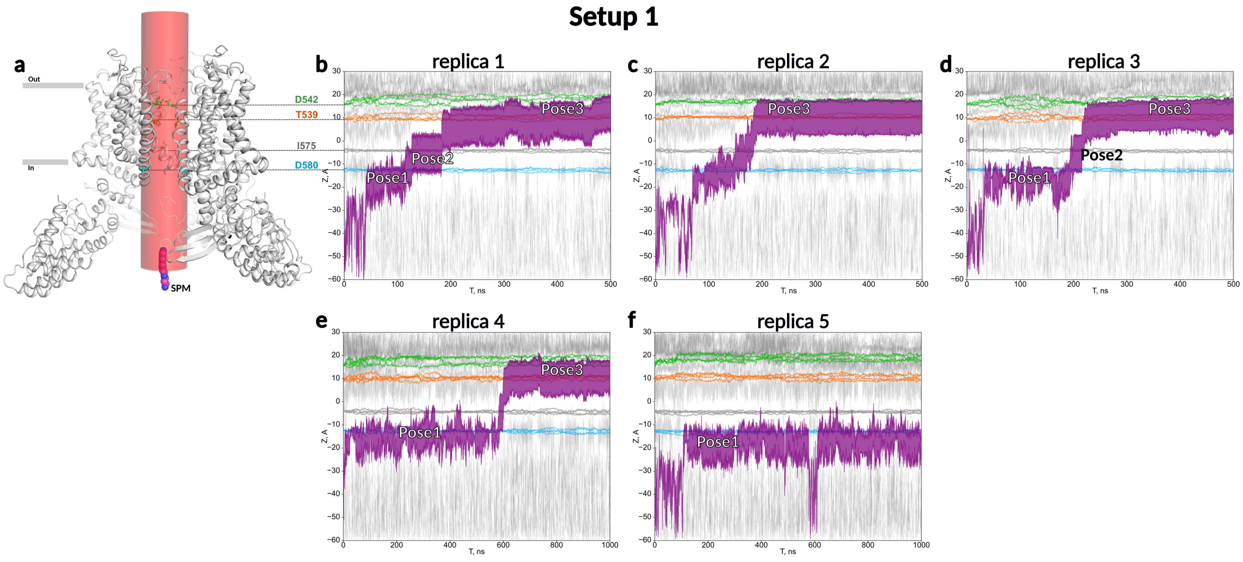

Supplementary Figure 6. Results of MD simulations for setup 1. a, Schematic representation of the initial configuration of the setup. Spermine is shown as magenta and blue spheres in its initial position below the pore entrance in bulk water. The red cylinder indicates the area within which spermine can move. hTRPV6$_{\mathrm{Open}}$ is shown in grey in a cartoon representation. **b-f**, Spermine movements during the MD trajectories. Magenta fillings represent spermine's positions along the pore axis, from the top to the bottom nitrogen atoms. Grey lines show the positions of Na^+. Colored lines correspond to the positions of C_α atoms of D580 (blue), I575 (dark grey), T539 (orange), and D542 (green); dashed lines project these residues onto the hTRPV6$_{\mathrm{Open}}$ structure. Poses 1-3 of spermine are marked on the plot. Panels **a** and **b** present the same data as in **Fig. 4a, b**.

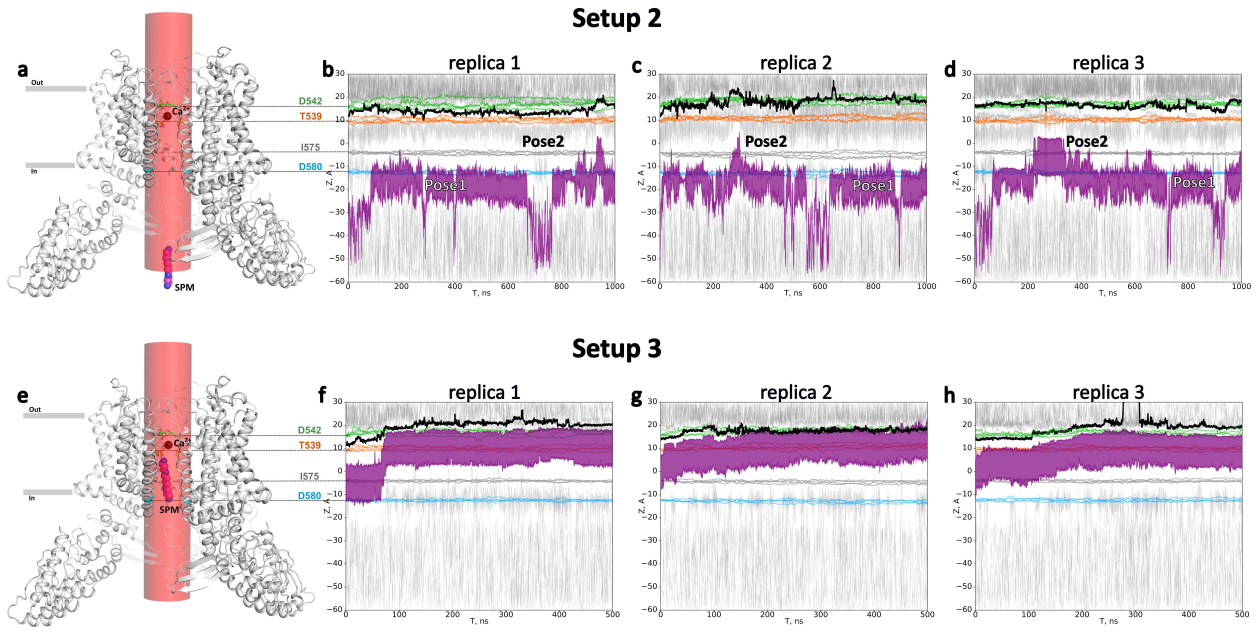

Supplementary Figure 7. Results of MD simulations for setups 2 (a-d) and 3 (e-h). Designations are the same as in **Supplementary Fig. 6**. The black sphere in panels **(a, e)** indicates the initial position of Ca^{2+}, while the black line in panels **(b–d, f–h)** shows its position during the simulations. In Setup 2 simulations, Na^{+} spontaneously occupied the central cavity of the pore between the spermine molecule and Ca^{2+}. Spermine did not knock off cations from the pore. Such behavior may be caused either by a higher affinity of Ca^{2+} to SF than spermine or by the formation of a high-energy barrier for spermine to reach Pose 3, which cannot be overcome within the timescale of our simulations. In the simulations with setup 3, the spermine molecule started from Pose 2, and Na^{+} could not penetrate the pore. In this case, spermine rapidly occupied Pose 3 in all three replicas, thus suggesting its stronger affinity towards the selectivity filter compared to Ca^{2+}.

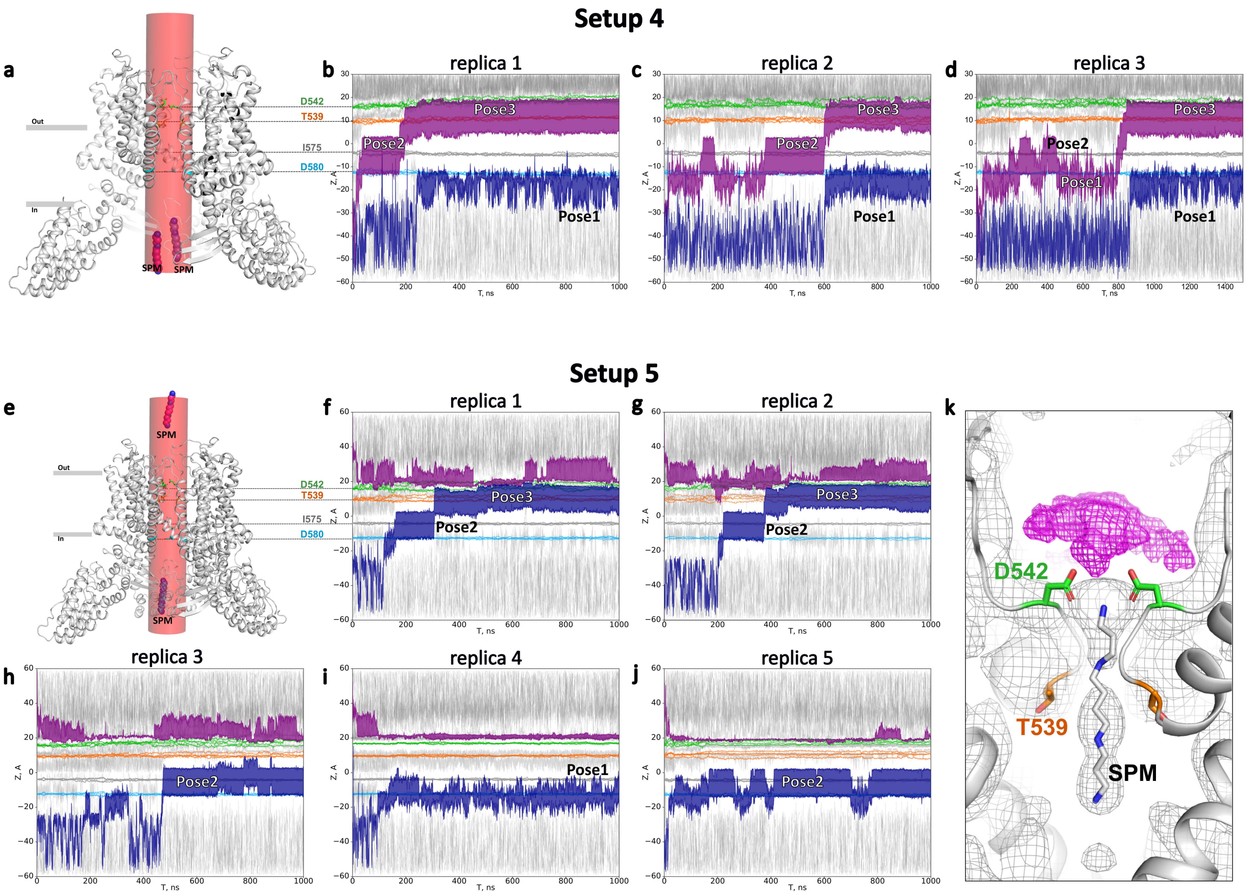

Supplementary Figure 8. Results of MD simulations for setups 4 (a-d) and 5 (e-j). Designations in the panels **(a-j)** are the same as in **Supplementary Fig. 6**. **k,** MD-averaged density of the spermine molecule in the extracellular vestibule of SF shown as a magenta mesh overlaid with cryo-EM non-protein densities within the pore (grey mesh). hTRPV6$_{Open}$ is shown in a cartoon representation, D542 and T539 are shown as colored sticks, spermine in Pose 3 – as gray sticks.

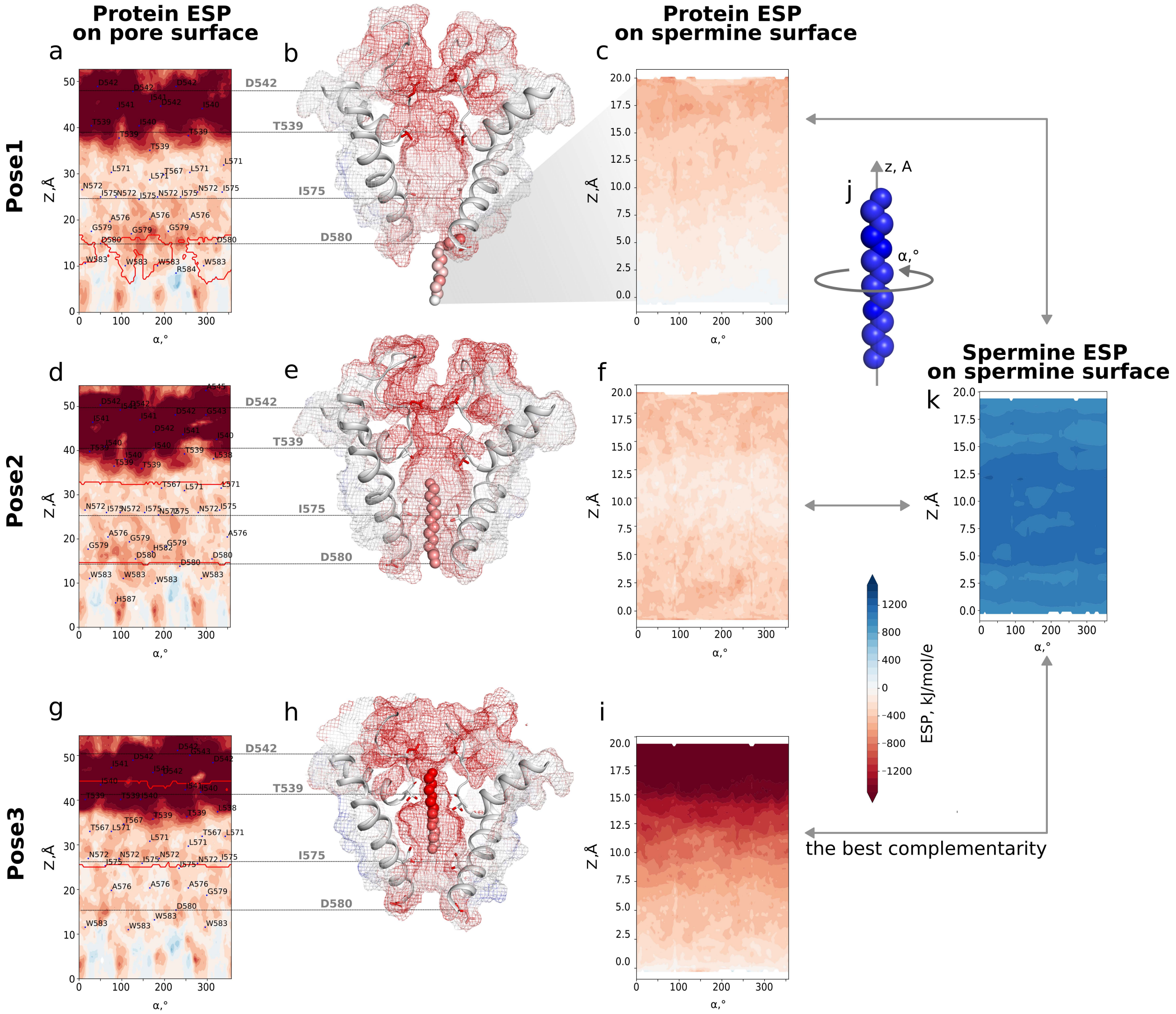

Supplementary Figure 9. Electrostatic potential (ESP) distribution within the pore when the spermine accommodates Pose 1 (a-c), Pose 2 (d-f), and Pose 3 (g-i). a, d, g, Cylindrical projection maps of protein-induced ESP distribution over the pore surface. The vertical axis (Z) represents the pore axis, and the horizontal axis (α) indicates the rotation angle around the pore axis. Essential pore-lining residues are labeled. **b, e, h,** Representative MD snapshots of the $hTRPV6_{Open}$ pore domain and the spermine molecule: $hTRPV6_{Open}$ is shown in grey cartoon representation (only two opposite subunits are shown). The mesh represents the $hTRPV6_{Open}$ surface and is colored according to its ESP. The spermine molecule is shown as spheres and colored according to the protein-induced ESP on its surface. **c, f, i,** Cylindrical projection maps of the protein-induced ESP distribution on the spermine surface with the coordinates represented schematically in **(j)**. **k,** Cylindrical projection maps of the spermine-induced ESP distribution on its own surface. In all maps, blue and red colors indicate surface areas with positive and negative potentials, respectively; the scale bar is shown on the right. ESP maps were calculated from MD trajectory data (Setup 1 - replica 1) (**Supplementary Fig. 6b**) and averaged over: 50-100 ns for Pose 1 (**b, c**), 130-170 ns – Pose 2 (**e, f**), 200-250 ns – Pose 3 (**h, i**). Analysis was performed using the Molecular Surface Topography (MST) tool within the CELL framework: https://model.nmr.ru/cell[103].

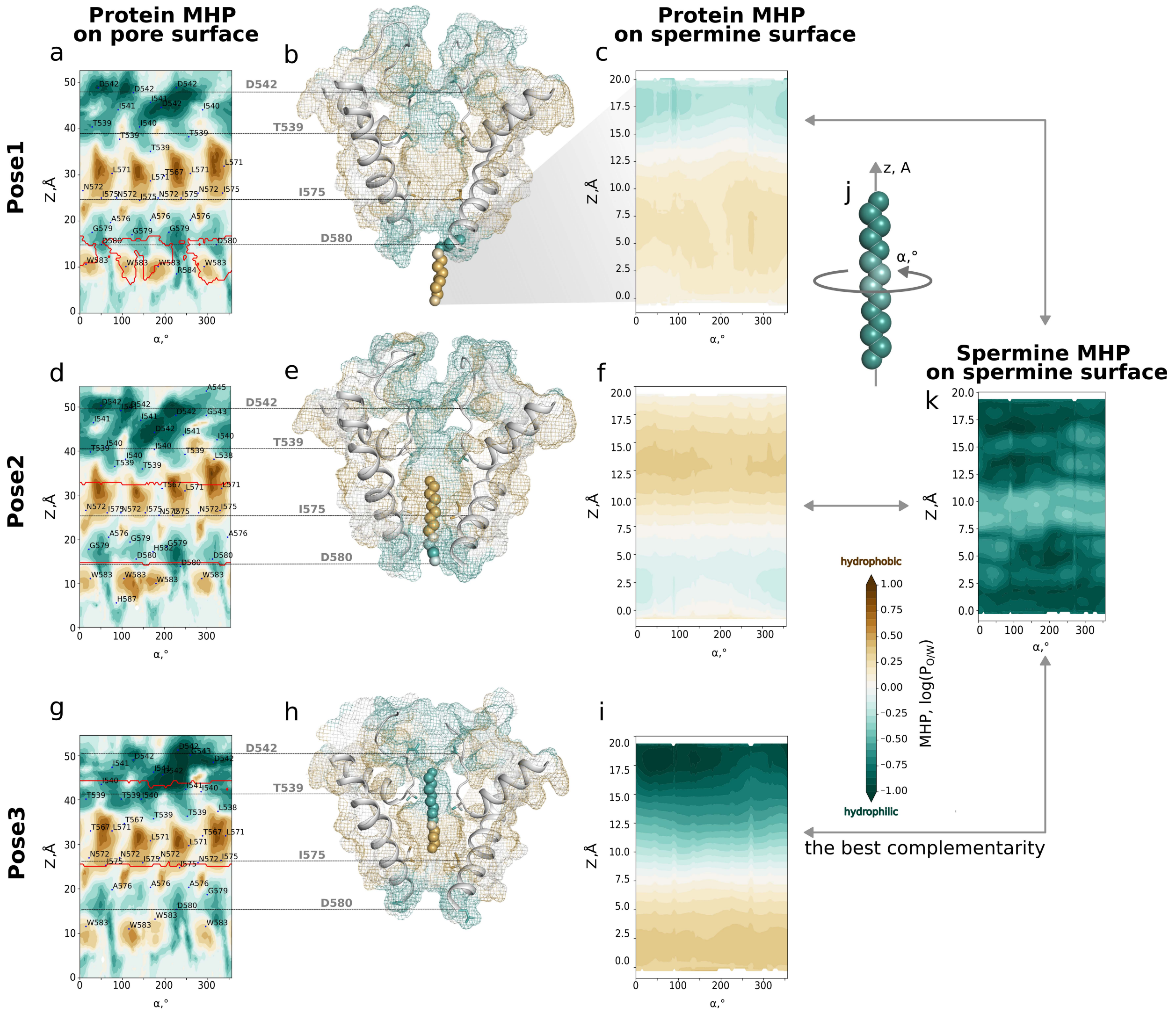

Supplementary Figure 10. Molecular hydrophobicity potential (MHP) distribution within the pore when the spermine accommodates Pose 1 (a-c), Pose 2 (d-f), and Pose 3 (g-i). a, d, g, Cylindrical projection maps of protein-induced MHP distribution over the pore surface. The vertical axis (Z) represents the pore axis, and the horizontal axis (α) indicates the rotation angle around the pore axis. Essential pore-lining residues are labeled. **b, e, h,** Representative MD snapshots of the $hTRPV6_{Open}$ pore domain and the spermine molecule: $hTRPV6_{Open}$ is shown in grey cartoon representation (only two opposite subunits are shown). The mesh represents the $hTRPV6_{Open}$ surface and is colored according to its MHP. The spermine molecule is shown as spheres and colored according to the protein-induced MHP on its surface. **c, f, i,** Cylindrical projection maps of the protein-induced MHP distribution on the spermine surface with the coordinates represented schematically in **(j)**. **k,** Cylindrical projection maps of the spermine-induced MHP distribution on its own surface. In all maps, brown and blue colors indicate hydrophobic and hydrophilic surface areas, respectively; the scale bar is shown on the right. MHP maps were calculated from MD trajectory data (Setup 1 - replica 1) (**Supplementary Fig. 6b**) and averaged over: 50-100 ns for Pose 1 (**b, c**), 130-170 ns – Pose 2 (**e, f**), 200-250 ns – Pose 3 (**h, i**). Analysis was performed using the Molecular Surface Topography (MST) tool within the CELL framework: https://model.nmr.ru/cell[103].

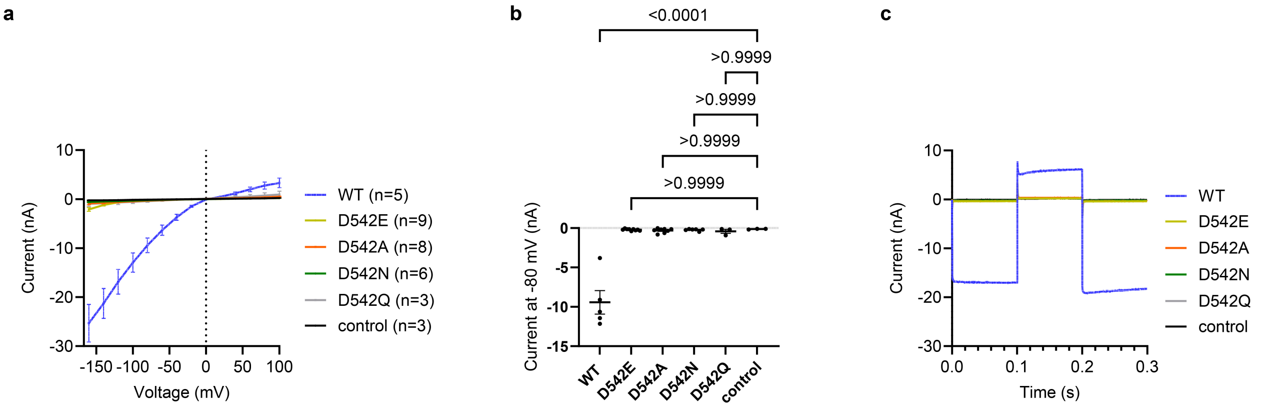

Supplementary Figure 11. Whole-cell currents of WT and mutant hTRPV6 variants expressed in HEK293 cells. a, Average voltage dependence of whole-cell currents recorded from untransfected HEK 293 cells (control) or HEK 293 cells expressing TRPV6 WT or TRPV6 with mutations at position D542 using the voltage step protocol shown in **Fig. 1** (data points for 199 ms of the step protocol). Data are shown as mean ± SEM, n is the number of experiments. **b**, Current amplitudes (mean ± SEM) measured at −80 mV from panel **a**. p values are shown for Holm-Šídák multiple comparisons test. Source data are provided. **c**, Representative currents elicited by the step protocol at +100 and −100 mV for WT and mutant TRPV6 variants.

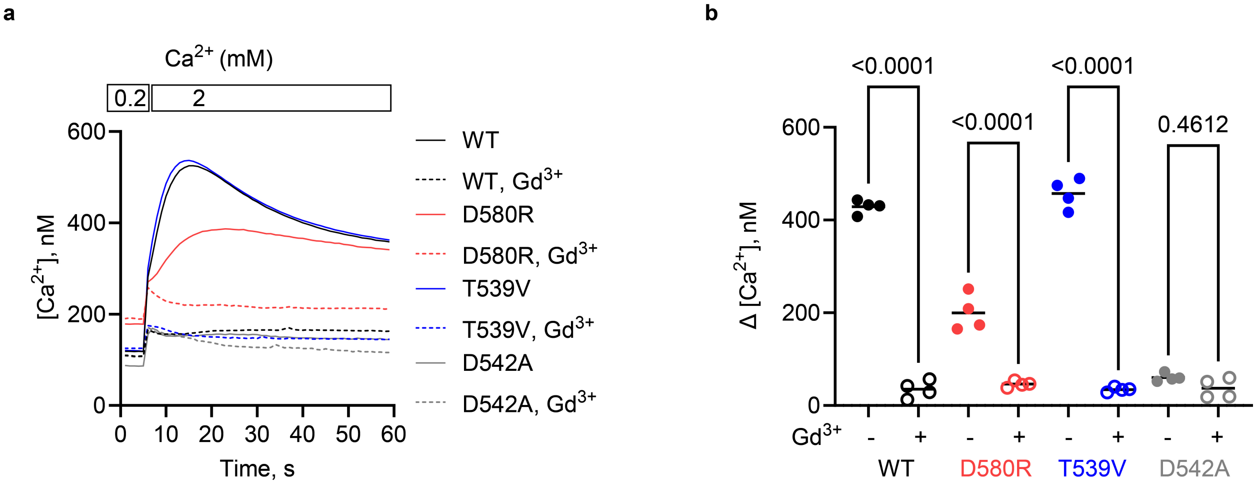

Supplementary Figure 12. TRPV6 inhibition by Gd^{3+}. a, Representative measurements of $[Ca^{2+}]_i$ in HEK 293 cells expressing hTRPV6-WT or mutants exposed to 0.2 or 2 mM extracellular Ca^{2+} in the absence or presence of 50 μM Gd^{3+}. **b**, Increases of $[Ca^{2+}]_i$ ($\Delta[Ca^{2+}]_i$) after 2 mM Ca^{2+} application calculated in experiments shown in (**a**). $\Delta[Ca^{2+}]_i$ were obtained in 4 independent measurements; p values are shown for Šídák's multiple comparisons test. Source data are provided.

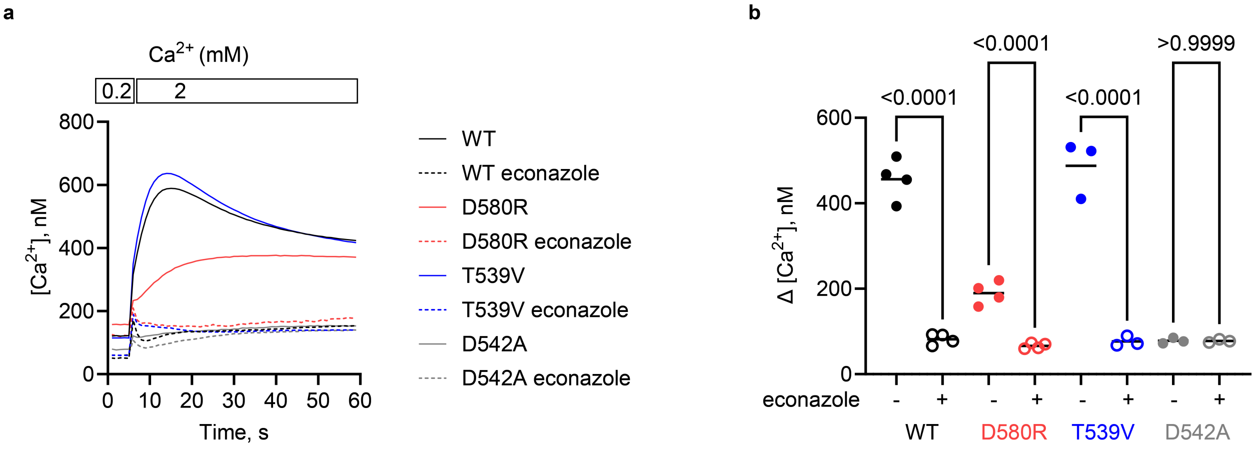

Supplementary Figure 13. TRPV6 inhibition by econazole. a, Representative measurements of $[Ca^{2+}]_i$ in HEK 293 cells expressing hTRPV6-WT or mutants exposed to 0.2 or 2 mM extracellular Ca^{2+} in the absence or presence of 20 μM econazole. **b**, Increases of $[Ca^{2+}]_i$ ($\Delta[Ca^{2+}]_i$) after 2 mM Ca^{2+} application calculated in experiments shown in (**a**). $\Delta[Ca^{2+}]_i$ were obtained in 3–4 independent measurements; p values are shown for Šídák's multiple comparisons test. Source data are provided.

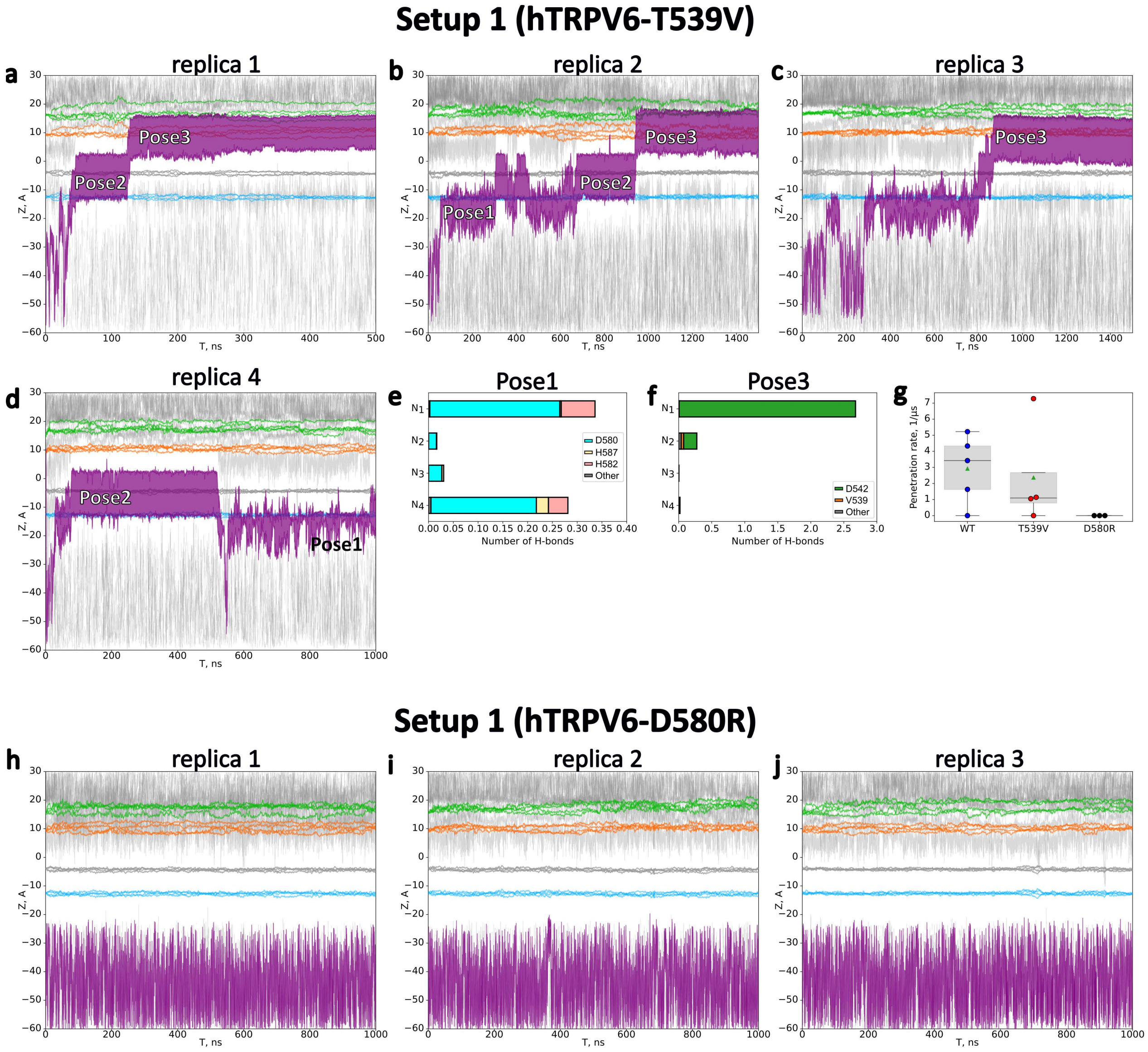

Supplementary Figure 14. Results of MD simulations for setup 1 for hTRPV6-T539V (a-g) and hTRPV6-D580R (h-j). Designations in panels **(a-d and h-j)** are the same as in **Supplementary Fig. 6**. **e, f**, Average number of h-bonds between the four nitrogen atoms of spermine (N_1-N_4) and TRPV6 residues in Pose 1 (**e**) and in Pose 3 (**f**) in MD simulations of hTRPV6-T539V. **g,** Rates of spermine penetration into Pose 3 (inverse time from the start of the simulation for Pose 3) are shown as box plots. Colored points represent the values obtained from MD trajectories with hTRPV6-WT (blue), hTRPV6-T539V (red), and hTRPV6-D580R (black). The average values are shown as green triangles. Zero rates indicate an absence of Pose 3 adoption. Source data are provided.

Supplementary Table 1. Cryo-EM data collection, refinement and validation statistics

**Structure**	hTRPV6$_{SPM}$
Preparation	cNW30/soybean lipids
EMDB accession code	EMD-76261
PDB accession code	12AG
**Data collection and processing**	
Magnification	105,000×
Voltage (kV)	300
Electron exposure ($e^{-}Å^{-2}$)	60
Defocus range (μm)	−0.75 to −2.0
Reported pixel size (Å)	0.788
Exposures (no.)	4,487
**Processing software**	
Motion correction	cryoSPARC v3.3
CTF estimation	cryoSPARC v3.3
Platform software for particle picking	cryoSPARC v3.3
Software for 2D/3D class. & Refinements	cryoSPARC v3.3
Symmetry imposed	C4
Initial particle images (no.)	2,191,044
Final particle images (no.)	14,018
Map resolution (Å)	3.48
FSC 0.143	
**Refinement**	
Initial models used (PDB code)	7S89
Model resolution (Å)	3.48
FSC threshold	0.143
Map sharpening *B* factor ($Å^2$)	−95.8
**Model composition**	
Non-hydrogen atoms	20,695
Protein residues	2,440
Ligands	30
Water	4
B factors ($Å^2$)	
Protein	140.91
Ligands	89.54
Water	7.24
R.m.s. deviations	
Bond lengths (Å)	0.007
Bond angles (°)	1.345
**Validation**	
MolProbity score	2.01
Clash score, all atoms	8.42
Poor rotamers (%)	1.32
Ramachandran plot	
Favored (%)	92.56
Allowed (%)	6.95
Disallowed (%)	0.49

Supplementary Table 2. MD simulation setups used in this study.

Setup number	Number of spermine molecules	Spermine starting position	Ca^{2+} starting position	Spermine final position	Number of replicas × simulation time (ns)	Figure of setup
1	1	intracellular	no	4 × Pose 3<br>1 × Pose 1	3 × 500<br>2 × 1000	Fig. 4a, Suppl. Fig. 6a
2	1	intracellular	SF	3 × Pose 1	3 × 1000	Suppl. Fig. 7a
3	1	Pose 2	SF	3 × Pose 3	3 × 500	Suppl. Fig. 7e
4	2	2 × intracellular	no	3 × (Pose 1 and Pose 3)	2 × 1000<br>1 × 1500	Suppl. Fig. 8a
5	2	1 × intracellular<br>1 × extracellular	no	2 ×(Pose 3 and extracellular)<br>2×(Pose 2 and extracellular)<br>1×(Pose 1 and extracellular)	5 × 1000	Suppl. Fig. 8e
1 (T539V)	1	intracellular	no	3 × Pose 3<br>1 × Pose 1	1 × 500<br>1 × 1000<br>2 × 1500	Suppl. Fig. 6a
1 (D580R)	1	intracellular	no	3 × bulk water	3 × 1000	Suppl. Fig. 6a
