# Supplementary material for: Open-channel block of human TRPV6 by polyamine spermine
Source: Nat Commun. 2026 May 27;17:4720. doi: 10.1038/s41467-026-73653-5 (PMC13216613; doi:10.1038/s41467-026-73653-5)
Supplement: Supplementary file 2 — Description of Additional Supplementary Files [file 41467_2026_73653_MOESM2_ESM.pdf]

Description of Additional Supplementary Files

File name: Supplementary Movie 1
Description: MD simulation of hTRPV6 block by intracellular spermine. Shown are frames of molecular dynamics (MD) simulation of $hTRPV6_{Open}$ embedded in a hydrated lipid membrane, approached by an intracellular spermine molecule, which is allowed to move freely within and near the channel pore (see Methods). Three metastable poses can be clearly distinguished throughout the stepwise binding pathway. Pose 1 is located at the intracellular entrance to the pore, where the positively charged groups of spermine form hydrogen bonds with D580. At this site, spermine is highly dynamic, frequently switching between two orientations, along the pore and transversal to the pore axis. In Pose 2, spermine is trapped in the central cavity and stretched along the pore axis. Its bottom nitrogen atom is located near the D580 residues but does not form hydrogen bonds with them. This pose likely arises due to the energetic barrier formed by cations near the selectivity filter, thus impeding further spermine penetration. When these cations are knocked off by spermine, spermine adopts Pose 3 inside the selectivity filter and forms h-bonds with D542 and T539.
